# High-density lipoprotein cholesterol for the prediction of mortality in cirrhosis with portal vein thrombosis: a retrospective study

**DOI:** 10.1186/s12944-019-1005-8

**Published:** 2019-03-30

**Authors:** Bo Gao, Jiangqiang Xiao, Ming Zhang, Feng Zhang, Wei Zhang, Jian Yang, Jian He, Yu Liu, Xiaoping Zou, Ping Xu, Yuzheng Zhuge

**Affiliations:** 1 Department of Gastroenterology and Clinical Nutrition, Nanjing Medical University Drum Tower Clinical Medical School, 321zhongshan road, Gulou District, Nanjing, 210008 China; 2 Department of Gastroenterology, Drum Tower Hospital, Nanjing University School of Medicine, Nanjing, China; 3 Department of Ultrasonography, Drum Tower Hospital, Nanjing University School of Medicine, Nanjing, China; 4 Department of Radiology, Drum Tower Hospital, Nanjing University School of Medicine, Nanjing, China; 5 Department of Gynecology and Obstetrics, The affiliated Obstetrics and Gynecology Hospital with Nanjing Medical University, Nanjing Maternity and Child Health Care Hospital, Nanjing, China; 6 Department of Gastroenterology and Hepatology, Nanjing Medical University Drum Tower Clinical Medical School, 321zhongshan road, Gulou District, Nanjing, 210008 China

**Keywords:** Cirrhosis, Portal vein thrombosis, High density lipoprotein cholesterol, Liver function, Prognosis

## Abstract

**Background:**

Lipid profiles disorders frequently occur in patients with chronic liver diseases, and the mortality of cirrhosis complicated with portal vein thrombosis (PVT) remains high. Research identifying simple and objective prognosis indicators for cirrhotic PVT has been limited. The aim of the present study was to investigate the association between lipid profiles and liver function, which may help predict the 1-year mortality in non-malignant cirrhosis with PVT.

**Methods:**

A retrospective cohort of 117 subjects with non-malignant cirrhotic PVT was conducted. The primary indicators of lipid profiles included triglyceride, cholesterol, high-density lipoprotein cholesterol (HDL-C) and low-density lipoprotein cholesterol. Correlations of lipid profiles with liver function tests, the Child-Turcotte-Pugh (CTP) score and the model for end-stage liver disease (MELD) score were investigated. The relationship between lipid profiles and 1-year mortality was assessed using the area under the receiver operating characteristic curves (AUROC). Logistic regression models were established to confirm the association between HDL-C and mortality.

**Results:**

The level of HDL-C was significantly decreased in non-survivors (*p* < 0.01) and patients with more severe liver damage stages (CTP *p* < 0.001; MELD *p* < 0.001). There was no significant difference in the HDL-C level among patients with different severities of PVT (*p* = 0.498). The level of HDL-C was positively correlated with albumin (*p* < 0.001, R = 0.438) and platelet (*p* = 0.022, R = 0.212) levels. The level of HDL-C was negatively correlated with bilirubin (*p* < 0.001, R = − 0.319), C-reactive protein (*p* < 0.001, R = − 0.342), the aspartate aminotransferase to alanine aminotransferase ratio (*p* < 0.0.1, R = − 0.237), the CTP score (*p* < 0.001, R = − 0.397) and the MELD score (*p* < 0.001, R = − 0.406). The 1-year mortality rate was 12.8%. The AUROC of HDL-C for the prediction of 1-year mortality in this population was 0.744 (*p* < 0.01, 95%CI 0.609–0.879). The level of HDL-C was independently associated with mortality by multivariate logistic regression models.

**Conclusions:**

The HDL-C level significantly decreases with the deterioration of liver function, which may serve as a potential indicator for the prognosis of non-malignant cirrhotic patients with PVT.

## Introduction

Liver cirrhosis can progress into a decompensated stage with a poor prognosis [1, 2]. Portal vein thrombosis (PVT) is one of the complications of decompensated cirrhosis, with an incidence rate of approximately 10 to 23% [3]. PVT is considered a negative prognostic indicator for cirrhosis with risk of portal hypertension [4–6]. The bleeding risk resulting from portal hypertension is nearly three-fold higher in patients with PVT than with cirrhosis alone [7]. The Child-Turcotte-Pugh (CTP) score and the model for end-stage liver disease (MELD) score are widely used to evaluate the severity of liver dysfunction and predict the outcomes of cirrhotic patients [3, 4]. However, these two score models have not been validated in cirrhosis patients complicated with portal vein thrombosis. The CTP scoring system consists of two subjective parameters including ascites and encephalopathy, which are prone to deviate between different evaluators [8]. The MELD score is determined by three laboratory test results (serum bilirubin, creatinine concentration and international normalized ratio-INR), while its drawback lies in the complexity of its calculation [8, 9]. Therefore, identification of certain objective indicator obtained from routine examinations that can provide a predictive value for patients with liver cirrhosis complicated by PVT is warranted.

The liver is an essential organ for lipid and lipoprotein synthesis, secretion and metabolism [10, 11]. Changes in liver function in chronic liver diseases could result in alterations of lipid profiles [12]. Previous studies have shown that lipid profile levels are important reflections of liver damage [12–14]. Habib et al. demonstrated that high-density lipoprotein cholesterol (HDL-C) was closely correlated with liver function [13]. Based on a multivariate logistic regression model, HDL-C was an independent predictor of transplant-free mortality in non-cholestatic cirrhotic veterans [13]. Kinueooi et al. suggested that, compared with healthy controls, the levels of triglyceride, cholesterol and HDL-C were significantly decreased in cirrhotic patients [15]. Moreover, HDL-C levels in hepatic carcinoma and metastatic liver cancer differed between survivors and non-survivors. Low-density lipoprotein cholesterol (LDL-C) level was also different between survival conditions in metastatic liver cancer [15]. To our knowledge, no research to date has investigated changes of lipid profiles in cirrhosis with portal vein thrombosis in the Chinese population. Thus, the present study aimed to investigate the correlation of lipid profiles with liver function and its role as a predictor of the 1-year mortality of non-malignant cirrhosis in patients with PVT.

## Patients and methods

### Patients

From May 2012 to December 2017, cirrhosis patients complicated with portal vein thrombosis admitted to our medical center were evaluated. Inclusion criteria were clear diagnosis of liver cirrhosis either by liver biopsy or confirmed by clinical presentations, routine liver function tests and medical imaging techniques; and clear evidence of thrombosis in the portal venous system, confirmed by Doppler ultrasonography or contrast-enhanced computer tomography (CT), including thrombosis in the main portal vein (MPV), left or right branches, the superior mesenteric vein (SMV) and the splenic vein (SV). Cavernous transformation of the portal vein due to thrombosis was also included. Exclusion criteria were patient age younger than 18 or older than 80 years old; malignant tumors; previous receipt of liver transplantation; serious hypertension; diabetes; cardiovascular diseases; cerebrovascular diseases; primary kidney diseases; use of lipid regulating drugs within half a year; use of glucocorticoid medicines within half a year; obesity with body mass index (BMI) (=kilogram of body weight /[height in meters]^2^) above 28 kg/m^2^; lack of complete medical records; loss to follow up. According to previously published criteria, the cut-off value for obesity in the Chinese population is 28 kg/m^2^ [16]. According to the principles of the Declaration of Helsinki, this retrospective study was approved by the Clinical Research Ethics Committee of the medical center. Written consent was waived due to the study’s observational nature.

### Clinical data collection

The records of all patients were retrospectively reviewed from our department’s prospectively collected database. Demographic information collected included age, sex, body mass index, etiology of cirrhosis, smoking history, drinking history, degree of PVT (thrombosis obstructed partially or completely of the portal venous system; cavernous transformation was considered as a special and severe type of PVT degree), extent of PVT (within MPV or its branches, extended to SMV or SV; cavernous transformation was also considered a special and severe type of PVT extension), degree of ascites, splenectomy history, prothrombotic disorders, patient’s treatment after admission, the use of anticoagulant medicines, indication for TIPS, and portal vein recanalization. Laboratory tests were performed with fasting blood samples once admitted. Laboratory indicators included the international standard ratio (INR), platelet count, white blood cell count (WBC), hemoglobin, serum bilirubin, albumin, alanine aminotransferase (ALT), aspartate aminotransferase (AST), C-reactive protein (CRP) and creatinine. Lipid profile indicators included triglyceride, cholesterol, high-density lipoprotein cholesterol and low-density lipoprotein cholesterol. Triglyceride, cholesterol and HDL-C were measured by a biochemistry analyzer (Abbott Laboratories, Abbott Park, USA). LDL-C was calculated by the Friedewald formula [17]. The calculation methods of CTP and MELD scoring system were previously described [18, 19]. The median of the MELD score in the entire group was used to stratify liver dysfunction. Patients were also categorized according to the degree of portal vein thrombosis obstruction to reflect the severity of portal vein thrombosis.

### Follow up

Patients were followed up by telephone at 1, 3, 6 months and 1 year after discharge from the hospital. The endpoint of follow up was the date of death from all causes of illness related to liver dysfunction, the date of liver transplantation or the date of one year from discharge from the hospital. According to transplant-free survival conditions, patients were divided into two groups (survivors and non-survivors).

### Statistical analysis

Data analysis was performed by SPSS for windows (version 20.0, IBM, USA). Continuous variables were expressed as the means± standard deviation (SD) if data were normally distributed and as the medians and range if data were skewed. Categorical variables were expressed by frequencies and proportions and compared by Chi-square test (χ2 -test) or Fisher’s exact test. Normally distributed continuous variables were compared by independent Student’s t-test or one-way analysis of variance. Skewed continuous variables were compared by Mann-Whitney U-test or Kruskal-Wallis test when appropriate. The correlation of triglyceride, cholesterol, HDL-C and LDL-C with CTP or MELD scores was evaluated by Pearson’s χ2 test. Area under the receiver operating characteristic curve (AUROC) was used to assess the predicting performance of lipid profiles, CTP and MELD score for 1-year mortality in this population. Cut-off values were obtained with the maximal Youden index (sensitivity + specificity-1). Logistic univariate and multivariate regression (backward method) models adjusting for factors potentially related to prognosis were used to calculate odds ratios (OR) and 95% confidence intervals (CI). A two-sided *p*-value < 0.05 was considered significantly different.

## Results

### Clinical characteristics of the study population

**Fig. 1:**
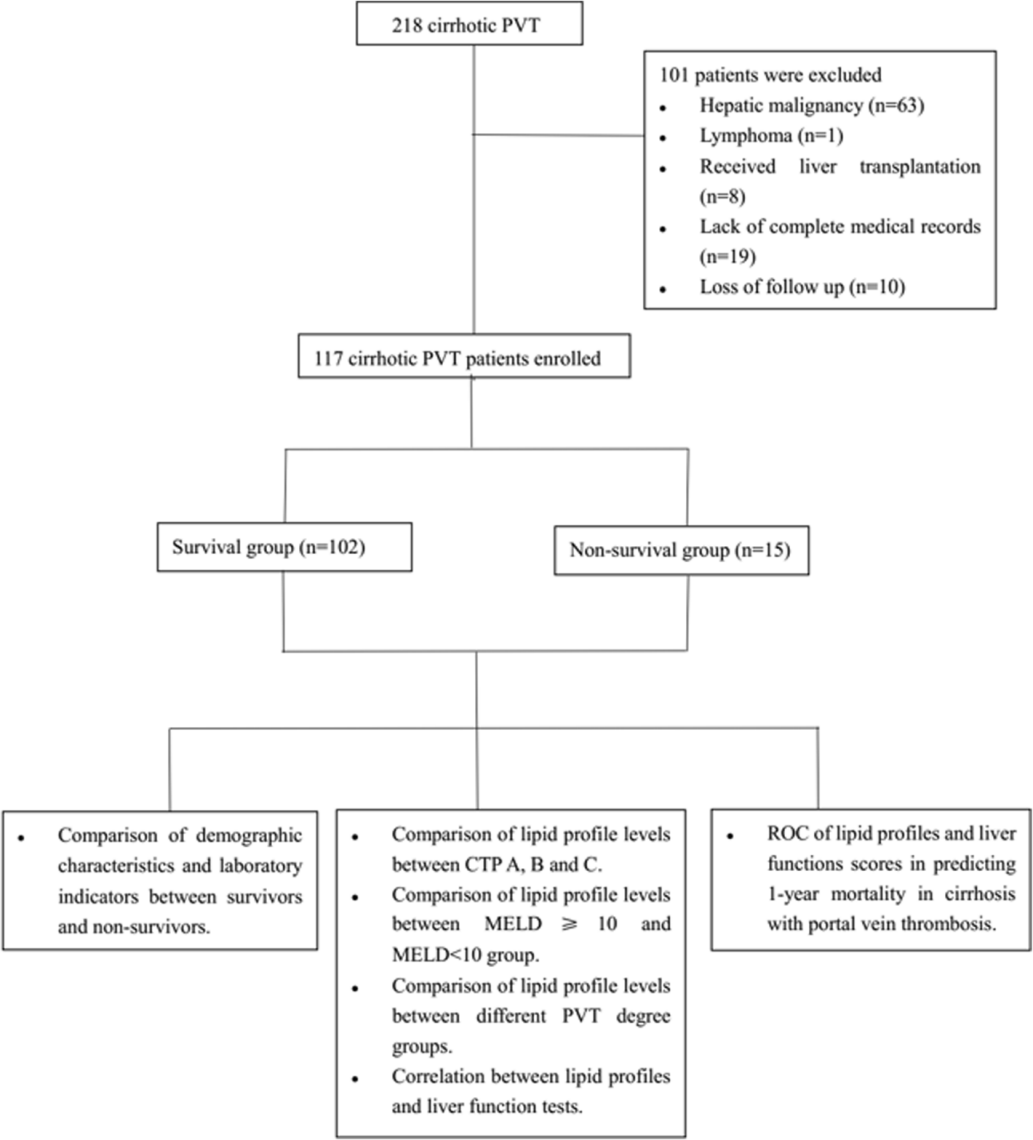
Flow diagram of the research

In total, 218 patients met the inclusion criteria. One-hundred and one patients were excluded: 63 patients were complicated with hepatic malignancy, 1 patient was complicated with lymphoma, 8 patients received liver transplantation before enrollment, 2 patients had coronary diseases, 5 patients used lipid regulation drugs within half a year, 1 patients had primary kidney disease, 2 patients suffered from severe diabetes, 11 patients had a lack of complete medical records, and 8 patients were lost of follow up. Finally, 117 patients were enrolled in the final cohort. Among them, fifteen died from all-causes of illness related to liver dysfunction within one year of admission. The 1-year mortality rate was 12.8%. The mean age of the entire group, survivors and non-survivors were 55.96 ± 11.90, 59.87 ± 12.31 and 55.38 ± 11.79, respectively. Male patients accounted for 62.4% of the overall population. The ratio of females in non-survivors surpassed that in survivors (53.3% vs. 35.3%, respectively). However, the difference was not statistically significant (*p* = 0.145). The BMI was not significantly different between the two groups (*p* = 0.440). The CTP and MELD scores were significantly higher in non-survivors than those in survivors (both *p* < 0.001). The most common etiology for cirrhosis was viral hepatitis. More than half of the population had thrombosis extended to SMV or SV. The percentages of patients suffering from complete and partial obstruction of thrombosis were 48.7 and 41% respectively. Ten percent of patients had cavernous transformation. The differences of degree and extent of PVT between survivors and non-survivors were not statistically significant (*p* = 0.655; 0.243). Among laboratory tests, levels of serum bilirubin (*p* = 0.011) and CRP (*p* < 0.01) were significantly higher in non-survivors. The level of albumin (*p* = 0.033) was significantly lower in non-survivors. Among the lipid profile indicators, only HDL-C levels were significantly decreased in non-survivors (*p* < 0.01) (Table 1). A flow diagram is shown in Fig. 1.

**Table 1:** Baseline characteristics

	Entire group *N* = 117	Non-survivors *N* = 15	Survivors *N* = 102	*p* value
Age (year)	55.96 ± 11.90	59.87 ± 12.31	55.38 ± 11.79	0.174
Gender				0.145
Male	73 (62.4)	7 (46.7)	66 (64.7)	
Female	44 (37.6)	8 (53.3)	36 (35.3)	
BMI (kg/m^2^)	23.60 ± 2.51	24.01 ± 1.82	23.54 ± 2.60	0.440
Child score (n, %)	7.39 ± 1.43	8.53 ± 1.64	7.23 ± 1.33	< 0.01^**^
A	33 (28.2)	2 (13.3)	31 (30.4)	
B	74 (63.2)	8 (53.3)	66 (64.7)	
C	10 (8.6)	5 (33.3)	5 (4.9)	
MELD score	9.75 ± 1.80	11.67 ± 1.72	9.67 ± 1.63	< 0.001^***^
Etiology (n, %)				0.067
Virus	53 (45.3)	5 (33.3)	59 (47.1)	
Others	64 (54.7)	10 (66.7)	43 (42.2)	
Schistosome	10	2	8	
PBC	4	2	2	
Alcoholic	9	1	8	
Autoimmune	6	1	5	
NAFLD	6	1	5	
Unknown	18	3	15	
Smoking history (n, %)	25 (21.4)	5 (33.3)	20 (19.6)	0.188
Drinking history (n, %)	14 (12.0)	3 (20)	11 (10.8)	0.257
Extent of PVT (n, %)				0.655
MPV or branches	44 (37.6)	4 (26.7)	40 (39.2)	
SMV/SV	61 (52.1)	9 (60)	52 (60)	
Cavernous	12 (10.3)	2 (13.3)	10 (9.8)	
Degree of PVT (n, %)				0.243
Partial	57 (48.7)	7 (46.7)	50 (49.0)	
Complete	48 (41.0)	6 (40)	42 (41.2)	
Cavernous	12 (10.3)	2 (13.3)	10 (9.8)	
Ascites (n, %)				0.073
Mild	23 (19.7)	2 (13.3)	21 (20.6)	
Moderate	32 (27.4)	1 (6.7)	31 (30.4)	
Severe	62 (52.9)	12 (80.0)	50 (49.0)	
Splenectomy history (n, %)	56 (47.9)	4 (26.7)	52 (51.0)	0.100
Prothrombotic (n, %)				0.052
Protein C deficiency	2 (1.7)	1 (6.7)	1 (1.0)	
Protein S deficiency	5 (4.3)	2 (13.3)	3 (2.9)	
JAK2V617F mutation	0 (0.0)	0 (0.0)	0 (0.0)	
Others	0 (0.0)	0 (0.0)	0 (0.0)	
Treatment (n, %)				0.065
TIPS	57 (48.7)	6 (40)	51 (50)	
EBL+ propranolol	34 (29.1)	2 (13.3)	32 (31.4)	
Others	26 (22.2)	7 (46.7)	19 (18.6)	
Anticoagulant (n, %)	83 (70.9)	13 (86.7)	70 (68.6)	0.225
Indication for TIPS (n, %)				0.893
Variceal bleeding	12 (12/57)	1 (1/6)	11 (11/51)	
Ascites	4 (4/57)	0 (0/6)	4 (4/51)	
PVT	6 (6/57)	1 (1/6)	5 (5/51)	
PVT and bleeding	35 (35/57)	4 (4/6)	31 (31/51)	
PVT progression (n, %)				0.131
Improved	19 (16.2)	4 (33.3)	15 (18.6)	
Stable	34 (29.1)	4 (26.7)	30 (29.4)	
Worsened	10 (8.6)	3 (20)	7 (23.6)	
Unknown	54 (46.2)	4 (26.7)	50 (49.0)	
Causes of death (n, %)				–
Liver failure	–	7 (46.7)	–	
GI bleeding	–	5 (33.3)	–	
Encephalopathy	–	3 (20)	–	
1-year mortality rate (%)	12.8%	–	–	–
Laboratory				
INR	1.3 (1,3.4)	1.26(1.12,1.66)	1.3 (1,3.4)	0.220
Platelet (*10^9/L)	111 (22,416)	95 (30,276)	125 (22,416)	0.835
WBC (*10^9/L)	4.2 (0.7,32.4)	5.1 (1.1,32.4)	4.2 (0.7,28)	0.264
Hemoglobin (g/L)	79 (43,152)	92 (56,152)	78.5 (43,148)	0.160
Bilirubin (umol/L)	18.6(3.9133.6)	26.2(8.9133.6)	17.6 (3.9116)	0.011^*^
Albumin (g/L)	32.34 ± 4.17	30.12 ± 5.18	32.62 ± 4.03	0.033^*^
ALT (U/L)	18.4(4.6162.4)	23.9 (8.1,44.9)	17.65(4.6162.4)	0.403
AST (U/L)	29.8(9.9230.1)	37.4 (19,114.3)	28.55(9.9230.1)	0.065
AST/ALT ratio	1.48 (0.63,6.6)	1.75(0.93,4.62)	1.47(0.63,6.6)	0.142
CRP (mg/L)	5.1 (0.2137.7)	21.2 (1.2,77.7)	4.4(0.2137.7)	< 0.01^**^
Creatinine (umol/L)	60 (32,210)	63 (37,111)	60 (32,210)	0.744
Lipid profile				
Triglyceride (mmol/L)	0.68(0.16,4.93)	0.83(0.43,1.29)	0.67(0.16,4.93)	0.095
Cholesterol (mmol/L)	3.04 (1.2,5.7)	2.75(1.66,4.63)	3.06 (1.2,5.7)	0.841
HDL-C (mmol/L)	0.81(0.14,1.92)	0.40(0.14,0.96)	0.83(0.16,1.92)	< 0.01^**^
LDL-C (mmol/L)	1.58(0.58,3.88)	1.60(0.66,3.02)	1.58(0.58,3.88)	0.708

^*^indicated for statistically significant with *p* < 0.05; ^**^ indicated for *p* value< 0.01;

^***^indicated for *p* value< 0.001; *NAFLD* nonalcoholic fatty liver disease, *TIPS* transjugular intrahepatic portosystemic shunts, *EBL* endoscopic band ligation, *GI* gastric intestinal

### Lipid profile indicators and CTP score

According to previously published criteria for grouping CTP score [19], patients were divided into three groups. Patients with 5 to 6 points were graded as group A, patients with 7 to 9 points were graded as groups B and patients with 10 to 15 points were graded as group C. Levels of triglyceride and LDL-C were not significantly different between groups. Cholesterol and HDL-C levels were significantly decreased with increased CTP score (*p* < 0.01 and *p* < 0.001, respectively) (Table 2).

**Table 2:** Comparison of lipid profile levels between CTP A, B and C

	CTP A *N* = 33	CTP B *N* = 74	CTP C *N* = 10	*p* value
Triglyceride (mmol/L)	0.77(0.16–1.88)	0.68(0.21–4.93)	0.57(0.31–1.03)	0.421
Cholesterol (mmol/L)	3.39(2.1–5.7)	2.86(1.44–5.47)	2.65(1.2–5.03)	< 0.01^**^
HDL-C (mmol/L)	1.08(0.43–1.85)	0.78(0.15–1.92)	0.40(0.14–1.25)	< 0.001^***^
LDL-C (mmol/L)	1.68(0.9–3.2)	1.47(0.61–3.88)	1.42(0.58–2.59)	0.089

^*^indicated for statistically significant with *p* < 0.05; ^**^ indicated for *p* value< 0.01^***^; indicated for *p* value< 0.001

### Lipid profile indicators and MELD score

Since no standard threshold point for MELD score was reached in consensus to classify liver function, we used the median score of our study group to stratify patients with different liver functions. The median MELD score of the overall patients was 10. Patients were divided into groups with MELD< 10 and MELD≥10. Levels of triglyceride were not statistically significant between the two groups. Cholesterol levels, both HDL-C and LDL-C levels were significantly lower in patients with MELD score ≥ 10 (all *p* < 0.001) (Table 3).

**Table 3:** Comparison of lipid profile levels between MELD≥10 and MELD< 10

	MELD< 10 *N* = 57	MELD≥10 *N* = 60	*p* value
Triglyceride (mmol/L)	0.71 (0.16–4.93)	0.66 (0.21–1.88)	0.545
Cholesterol (mmol/L)	3.37 (1.99–5.7)	2.74 (1.2–4.78)	< 0.001^**^
HDL-C (mmol/L)	0.97 (0.36–1.85)	0.66 (0.14–1.92)	< 0.001^**^
LDL-C (mmol/L)	1.76 (0.69–3.88)	1.32 (0.58–3.02)	< 0.001^**^

^*^indicated for statistically significant with *p* < 0.05; ^**^ indicated for *p* value< 0.01; ^***^ indicated for *p* value< 0.001

### Lipid profile indicators and PVT severity

The triglyceride (*p* = 0.220), cholesterol (*p* = 0.344), HDL-C (*p* = 0.498) and LDL-C (*p* = 0.734) levels were not significantly different among portal vein thrombosis severity groups (Table 4).

**Table 4:** Comparison of lipid profile levels between different PVT severity degree

	Partial *N* = 57	Complete *N* = 48	Cavernous *N* = 12	*p* value
Triglyceride (mmol/L)	0.64(0.16–1.87)	0.78(0.24–4.93)	0.77(0.49–1.38)	0.220
Cholesterol (mmol/L)	2.85(1.44–5.3)	3.12(1.2–5.7)	3.42(2.09–4.63)	0.344
HDL-C (mmol/L)	0.80(0.14–1.84)	0.81(0.15–1.92)	0.91(0.54–1.71)	0.498
LDL-C (mmol/L)	1.47(0.62–3.2)	1.61(0.58–3.88)	1.79(0.85–3.02)	0.734

^*^indicated for statistically significant with *p* < 0.05; ^**^ indicated for *p* value< 0.01; ^***^ indicated for *p* value< 0.001

### Correlations of lipid profile indicators with liver function tests

The correlations of lipid profile indicators (triglyceride, cholesterol, HDL-C and LDL-C) with liver function biomarkers (INR, bilirubin, albumin, CRP, platelet, AST/ALT ratio) and liver dysfunction scoring results (CTP score and MELD score) were assessed by Pearson χ2 test. A *p*-value < 0.05 was considered to be a significant correlation between parameters. With *p*-value < 0.05, the closer the absolute value of R was to 1, the greater the degree of correlation [20]. Triglycerides were not correlated with any of the liver function biomarkers and scores. Cholesterol levels were significantly correlated with the INR (*p* = 0.041, R = − 0.189), albumin levels (*p* < 0.001, R = 0.361), platelet levels (*p* < 0.01, R = 0.268), the CTP score (*p* < 0.01, R = − 0.314) and the MELD score (*p* < 0.001, R = − 0.387). LDC-C levels were also significantly correlated with liver function, including albumin levels (*p* < 0.001, R = 0.348), platelet levels (*p* = 0.023, R = 0.210), the CTP score (*p* < 0.01, R = − 0.259) and the MELD score (*p* < 0.001, R = − 0.353). Notably, HDL-C was significantly correlated with all of the liver function biomarkers except for INR. HDL-C was negatively correlated with bilirubin (*p* < 0.001, R = − 0.319), CRP (*p* < 0.001, R = − 0.342), the AST/ALT ratio (*p* < 0.0.1, R = − 0.237), the CTP score (*p* < 0.001, R = − 0.397) and the MELD score (*p* < 0.001, R = − 0.406), which indicates that HDL-C levels are lower with increases of such indicators. The HDL-C levels were positively correlated with albumin (*p* < 0.001, R = 0.438) and platelet (*p* = 0.022, R = 0.212) levels, indicating that HDL-C levels are higher with increased albumin and platelet levels (Table 5).

**Table 5:** Correlation between lipid profile levels and liver function tests

	Correlation	Triglyceride	Cholesterol	HDL-C	LDL-C
INR	R	− 0.064	− 0.189	− 0.115	− 0.176
	*p*	0.490	0.041^*^	0.219	0.058
Bilirubin	R	− 0.046	−0.010	− 0.319	−0.074
	*p*	0.624	0.918	< 0.001^***^	0.425
Albumin	R	0.067	0.361	0.438	0.348
	*p*	0.473	< 0.001^**^	< 0.001^***^	< 0.001^***^
CRP	R	0.029^*^	− 0.160	−0.342	− 0.166
	*p*	0.756	0.084	< 0.001^***^	0.074
PLT	R	0.103	0.268	0.212	0.210
	*p*	0.267	< 0.01^**^	0.022^*^	0.023^*^
AST/ALT ratio	R	−0.085	− 0.207	−0.237	− 0.090
	*p*	0.363	0.025^*^	< 0.01^**^	0.333
CTP	R	−0.156	−0.314	− 0.397	−0.259
	*p*	0.093	< 0.01^**^	< 0.001^***^	< 0.01^**^
MELD	R	−0.071	− 0.387	− 0.406	− 0.353
	*p*	0.446	< 0.001^***^	< 0.001^***^	< 0.001^***^

^*^indicated for statistically significant with *p* < 0.05; ^**^ indicated for *p* value< 0.01; ^***^ indicated for *p* value< 0.001

### Lipid profile and 1-year mortality

**Fig. 2:**
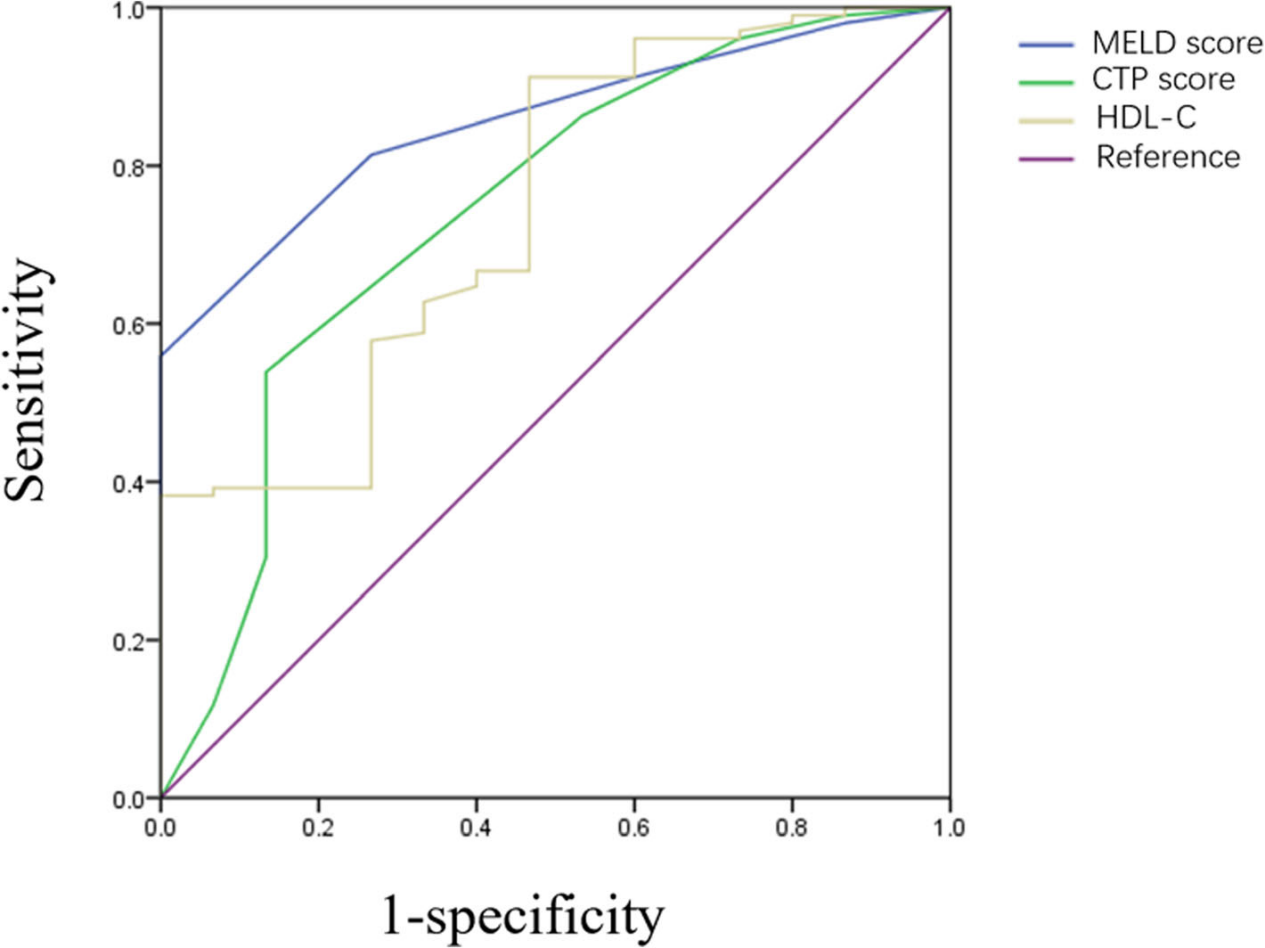
Receiver operating curves of HDL-C, CTP score and MELD score. Legends of Fig. 2 The ROC of HDL-C in the figure was logarithmic transformed. The AUROC curve shows the ability of HDL-C, CTP score and MELD score in predicting mortality for cirrhotic PVT. The AUROC of HDL-C was 0.744 (95%CI 0.609–0.879); The AUROC of CTP score was 0.743 (95%CI 0.597–0.890); The AUROC of MELD score was 0.855 (95%CI 0.778–0.932). The results indicated that HDL-C, CTP score and MELD score had satisfactory performance in predicting mortality of cirrhotic PVT

The sensitivity and specificity of lipid profile indicators as predictors of 1-year mortality in cirrhosis patients with portal vein thrombosis were assessed by receiver operating characteristic curves. Among lipid profile indicators, only AUROC of HDL-C was statistically significant in predicting 1-year mortality (AUROC = 0.744, *p* < 0.01, 95%CI 0.609–0.879). Triglyceride, cholesterol and LDL-C levels were unable to predict mortality in the present study. We also evaluated the predictive accuracy performance of CTP score and MELD score. Consistent with traditional opinion, both CTP and MELD score could predict 1-year mortality in cirrhosis with portal vein thrombosis. The AUROCs of CTP and MELD scores were 0.743 (*p* < 0.01, 95%CI 0.597–0.890) and 0.855 (*p* < 0.001, 95%CI 0.778–0.932), respectively. Obtained by the maximal Youden index (sensitivity + specificity-1), the best cut-off value of HDL-C was 0.42 mmol/L, with a sensitivity of 91.2% and specificity of 53.3%. The results of the AUROC of lipid profile indicators and scoring systems are summarized in Table 6. The ROCs of HDL, CTP and MELD are shown in Fig. 2.

**Table 6:** ROC of lipid profiles as predictors for 1-year mortality in cirrhotic PVT

	AUROC	SE	*p* value	95% confidence interval	
				Lower	Upper
Triglyceride	0.634	0.066	0.095	0.505	0.763
Cholesterol	0.533	0.084	0.681	0.368	0.698
HDL-C	0.744	0.069	< 0.01^**^	0.609	0.879
LDL-C	0.503	0.090	0.974	0.327	0.678
CTP score	0.743	0.075	< 0.01^**^	0.597	0.890
MELD score	0.855	0.039	< 0.001^***^	0.778	0.932

^*^indicated for statistically significant with *p* < 0.05; ^**^ indicated for *p* value< 0.01;

^***^indicated for *p* value< 0.001; *AUROC* area under receiver operating curve, *SE* standard error

### Univariate and multivariate logistic regression analysis

HDL-C level was stratified by a cut-off value of 0.42 mmol/L. In model 1, CTP score, MELD score and HDL-C grade showed significant differences by univariate analysis. After adjusting for three indicators, MELD score (OR = 1.636, 95%CI 1.163–2.302, *p* < 0.01) and HDL-C (OR = 4.597, 95%CI 1.089–19.398, *p* = 0.038) were associated with mortality. Age and gender were adjusted in model 2, and only HDL-C (OR = 12.573, 95%CI 3.483–45.382, *p* < 0.001) was an independent factor for morality. CRP, albumin and bilirubin levels, which were significantly different between survivors and non-survivors, were adjusted in model 3. Only HDL-C grade (OR = 7.772, 95%CI 1.662–35.878, *p* < 0.01) was independently associated with mortality. In models 4, 5 and 6, HDL-C grade was a prognostic factor independent of etiology, treatment strategy and PVT degree (OR = 11.545, 95%CI 3.265–40.858, *p* < 0.001; OR = 9.98, 95%CI 2.836–35.117, *p* < 0.001; OR 14.76, 95%CI 3.973–54.832, *p* < 0.001, respectively) (Table 7).

**Table 7:** Logistic regression analysis of predictors for 1-year mortality

	Univariate analysis		Multivariate analysis	
	OR (95%CI)	*p* value	OR (95%CI)	*p* value
Model 1				
CTP score	1.997(1.286–3.099)	< 0.01		
MELD score	1.898(1.354–2.661)	< 0.001	1.636(1.163–2.302)	< 0.01^**^
HDL-C				
≥ 0. 42 mmol/L	Reference		Reference	
< 0. 42 mmol/L	11.81(3.474–40.144)	< 0.001	4.597(1.089–19.398)	0.038^*^
Model 2				
Age (year)	1.034(0.985–1.084)	0.175		
Sex				
Male	Reference			
Female	0.477(1.600–1.423)	0.185		
HDL-C				
≥ 0. 42 mmol/L	Reference		Reference	
< 0. 42 mmol/L	11.81(3.474–40.144)	< 0.001	12.573(3.483–45.382)	< 0.001^***^
Model 3				
CRP mg/L	1.020(1.001–1.039)	0.042		
Albumin g/L	1.027(1.003–1.052)	0.028		
Bilirubin umol/L	0.859(0.749–0.986)	0.030		
HDL-C				
≥ 0. 42 mmol/L	Reference		Reference	
< 0. 42 mmol/L	11.81(3.474–40.144)	< 0.001	7.722(1.662–35.878)	< 0.01^**^
Model 4				
Etiology				
Viruses	0.31(0.098–0.973)	0.045		
Others	Reference			
HDL-C				
≥ 0. 42 mmol/L	Reference		Reference	
< 0. 42 mmol/L	11.81(3.474–40.144)	< 0.001	11.548(3.265–40.845)	< 0.001^***^
Model 5				
Treatment				
TIPS	0.170(0.032–0.902)	0.037		
EBL	0.319(0.095–1.072)	0.065		
Anticoagulant	Reference			
HDL-C				
≥ 0. 42 mmol/L	Reference		Reference	
< 0. 42 mmol/L	11.81(3.474–40.144)	< 0.001	9.98(2.836–35.117)	< 0.001^***^
Model 6				
PVT degree				
Partial	0.714(0.125–4.080)	0.705		
Complete	0.700(0.126–3.878)	0.683		
Cavernous	Reference			
HDL-C				
≥ 0. 42 mmol/L	Reference		Reference	
< 0. 42 mmol/L	11.81(3.474–40.144)	< 0.001	14.760(3.973–54.832)	< 0.001^***^

^*^indicated for statistically significant with *p* < 0.05; ^**^ indicated for *p* value< 0.01;

^***^indicated for *p* value< 0.001; *OR* odds ratio, *CI* confidence interval

## Discussion

The present study revealed that HDL-C is significantly correlated with liver function tests and liver function scores. Levels of HDL-C decreased significantly with the deterioration of liver function. Cholesterol and LDL-C levels also correlated with liver function indicators. These results were largely in line with a previous study conducted by Habib et al. [13]. Habib et al. found that HDL-C, but not cholesterol or LDL-C, was strongly associated with albumin, bilirubin, INR and MELD score. By logistic regression analysis, HDL-C was identified as an independent prognostic factor for 6- and 12-month mortality in non-cholestatic cirrhosis. Chrostek et al. [12] suggested that both HDL-C and LDL-C can act as biomarkers of the severity of liver dysfunction in non-alcoholic cirrhosis. In addition, we failed to find significant differences in triglyceride between groups with different levels of liver damage. In contrast, Jiang et al. [14] reported that the combination of triglyceride and MELD score can predict the mortality of decompensated liver cirrhosis. The discrepancy of these results may lie in the different ethnicities, etiologies and severity of liver damage included [13].

Chronic liver diseases affect liver synthesis function, leading to hypo-cholesterolemia and hypolipidemia [13, 21]. Moreover, abnormal changes of lipoprotein composition, metabolism and function are usually detected in patients with liver diseases [21]. In our research, cholesterol and HDL-C levels decreased significantly with the deterioration of liver function as assessed by CTP and MELD scores, and the level of LDL-C decreased along with the decline of MELD score. Among them, HDL-C was the most remarkable indicator. Furthermore, Wolf et al. found that HDL-C levels were elevated upon robust regeneration of the liver tissues after liver transplantation [22]. The odds ratio for segmental graft dysfunction was 0.61 for every 1 mg/dL increase of HDL-C level [22]. A previous study also concluded that HDL-C level could recover to normal after liver transplantation [23]. Therefore, HDL-C levels are associated with the degree of liver regeneration and function. Trieb et al. demonstrated that the level of HDL-C was reduced with liver damage severity, and the composition of HDL-C was also altered. The efflux capacity of HDL-C can be used to predict risks in cirrhosis patients [21]. Moreover, nutrition state deterioration usually occurs in the decompensated stage of liver diseases [24, 25]. Inadequate caloric and protein intake and progressive depletion of skeletal muscle observed in patients with chronic liver disorders lead to insufficient synthesis of lipoproteins and cholesterols. However, whether improving HDL-C via dietary supplement and exercise can contribute to optimizing liver function remains unclear and warrants further investigation.

Recent studies have focused on the anti-inflammatory effects of HDL-C [26, 27]. HDL-C promotes cholesterol efflux in macrophage foam cells, thereby preventing lipid secretion of proinflammatory cytokines [26]. In an animal model [27], the consumption of baru seeds to improve HDL-C was found to prevent iron-induced oxidative stress in rats. In our study, we revealed that HDL-C was negatively correlated with C-reactive protein, which somewhat reflects its anti-inflammatory effects. Interestingly, Marco van der Stoep et al. indicated that [28] HDL-C can act as a modulator of platelet and coagulation in venous thrombosis. Deguchi et al. [29] found significantly lower levels of HDL-C in deep vein thrombosis and pulmonary embolism patients. The same result was also concluded in a meta-analysis by Ageno et al. [30]. Using in vitro experiments [31–33], researchers discovered that high density lipoprotein serves as a modulator of both intrinsic and extrinsic coagulation cascades. HDL activates protein C, which is a key factor in the anti-coagulation pathway and suppresses thrombin generation [34, 35]. However, the present study did not find significant difference in HDL-C levels between groups with varying degrees of portal vein thrombosis. The correlation between HDL-C and portal vein thrombosis needs to be further studied.

It is generally acknowledged that cirrhosis patients complicated with PVT suffer from a poorer prognosis. [3]. Child-Turcotte-Pugh and model for end-stage liver disease allocating systems aid in the risk stratification and selection of priorities for progressive treatment [5, 8]. However, their efficacy in evaluating patients with PVT remains controversial. Although our study demonstrated that both CTP and MELD scores had the ability to predict the prognosis of cirrhosis with PVT, the two score methods have shortcomings. The CTP allocation system is comprised of two subjective indicators (degree of ascites and staging of hepatic encephalopathy), which may bring bias between different evaluators and affect its final accuracy. Albumin level, which is an important indicator in the CTP system, is prone to be affected by human serum albumin infusion in the clinic. The MELD allocation system is more objective than CTP; however, its limitation lies in the complexity of its calculation. Our study proved a significant correlation between HDL-C and liver function. Furthermore, HDL-C was also identified to have a predictive ability for the 1-year prognosis of cirrhotic patients with PVT. HDL-C levels were easy to obtain from biochemistry tests and were less affected by clinical therapies than albumin. We propose that HDL-C level should be included in routine examinations for cirrhosis patients, especially for those complicated with PVT.

There were several limitations in this study. First, it was based on a retrospective design, which may have led to certain biases [36]. Second, the present study contained a small sample size. However, considering the incidence of non-malignant liver cirrhosis and its complications, a large sample size study was difficult to conduct. Third, because this study only included subjects of Asian ethnicity, the conclusions might not be generalizable to other ethnic populations. A multicenter study design including a comparatively larger number of patients should be applied to validate our conclusions in the future.

## Conclusion

We conclude that HDL-C levels decreased with the deterioration of liver function. Importantly, HDL-C might be an independent determinant for the prognosis of cirrhosis patients with PVT. As decompensated cirrhosis with PVT is difficult to treat and could increase the burden on society with adverse long-term prognosis and increased morbidity, early screening and assessment for the risk of liver dysfunction must be considered.

## Abbreviations

ALT: Glutamic pyruvic transaminase
AST: Glutamic pyruvic aminotransferase
AUROC: Area under receiver operating characteristic curve
BMI: Body mass index
CI: Confidence interval
CRP: C-reactive protein
CTP: Child--Turcotte-Pugh
EBL: Endoscopic band ligation
GI: Gastric intestinal
HDL-C: High density lipoprotein cholesterol
INR: International standard ratio
LDL-C: Low density lipoprotein cholesterol
MELD: Model for end-stage liver disease
MPV: Main portal vein
OR: Odds ratio
PVT: Portal vein thrombosis
SMV: Superior mesenteric vein
SV: Splenic vein
TIPS: Transjugular intrahepatic portosystemic shunts
WBC: White blood cells
